# Hypothalamic Glial-to-Neuronal Signaling during Puberty: Influence of Alcohol

**DOI:** 10.3390/ijerph8072894

**Published:** 2011-07-14

**Authors:** Vinod K. Srivastava, Jill K. Hiney, W. Les Dees

**Affiliations:** Department of Veterinary Integrative Biosciences, College of Veterinary Medicine, Texas A&M University, College Station, TX 77843-4458, USA; E-Mails: vsrivastava@cvm.tamu.edu (V.K.S); jhiney@cvm.tamu.edu (J.K.H.)

**Keywords:** alcohol, puberty, transforming growth factor-α, glia, RPTPβ

## Abstract

Mammalian puberty requires complex interactions between glial and neuronal regulatory systems within the hypothalamus that results in the timely increase in the secretion of luteinizing hormone releasing hormone (LHRH). Assessing the molecules required for the development of coordinated communication networks between glia and LHRH neuron terminals in the basal hypothalamus, as well as identifying substances capable of affecting cell-cell communication are important. One such pathway involves growth factors of the epidermal growth factor (EGF) family that bind to specific erbB receptors. Activation of this receptor results in the release of prostaglandin-E_2_ (PGE_2_) from adjacent glial cells, which then acts on the nearby LHRH nerve terminals to elicit release of the peptide. Another pathway involves novel genes which synthesize adhesion/signaling proteins responsible for the structural integrity of bi-directional glial-neuronal communication. In this review, we will discuss the influence of these glial-neuronal communication pathways on the prepubertal LHRH secretory system, and furthermore, discuss the actions and interactions of alcohol on these two signaling processes.

## 1. Introduction

The hypothalamic region of the brain plays an important role in synchronizing events leading to the activation of the mammalian puberty. This process requires the interactive participation of both glial and neuronal regulatory circuitries that serve to control the secretion of luteinizing hormone-releasing hormone (LHRH) neurons [1,2]. The secretory activity of LHRH neurons is triggered by several transsynaptic inputs of both inhibitory and excitatory nature [1,3,4]. The decreased release of inhibitory neurotransmitters such as gamma amino butyric acid and the opiod peptides [5,6] as well as the increased release of excitatory neurotransmitters such as excitatory amino acids [7,8], transforming growth factor alpha (TGFα) [9], insulin-like growth factor-1 [10,11], and the kisspeptins [12] are capable of initiating the cascade of events leading to increased LHRH secretion at puberty. The glial cells within the medial basal hypothalamus (MBH) are key components of the central regulatory system that facilitate prepubertal LHRH release via pathways initiated by growth factors and cell adhesion molecules that act on LHRH neuron terminals to stimulate their secretory activity [13,14].

Growth factors of glial origin are important because they are intimately involved in glial-neuronal signaling processes by which the glial cells, through their close association with LHRH nerve terminals in the MBH, regulate LHRH secretion during puberty in rodents [13,15] and primates [16]. Growth factors such as basic fibroblast growth factor (bFGF) [17,18], transforming growth factor β (TGF-β) [19] and IGF-1 [10,20] have been shown to act directly on LHRH neurons to facilitate LHRH release. In contrast to these growth factors, members of the epidermal growth factor (EGF) family, including EGF itself, TGFα, and the neuregulins act indirectly on LHRH release through specific erbB receptors that have an extracellular ligand binding domain linked to a cytoplasmic domain containing tyrosine kinase activity [14,21,22]. While all of these peptides can influence LHRH release, a portion of this review will concentrate specifically on EGF/TGFα influences, since they have been shown to play a pivotal role in the glial control of neuronal LHRH secretion at the time of puberty.

During the past decade, evidence has accumulated suggesting LHRH secretory activity is also modulated by a specific glial-neuronal gene family which synthesizes adhesion/signaling proteins involved in the functional and structural integrity of bi-directional glial-neuronal communications. In this regard, various glial-neuronal adhesion genes have been identified within the hypothalamus which are not only involved in adhesive interactions, but also mediate intracellular signaling cascades that are critical for pubertal development [23,24]. The genes of this family include glial receptor protein tyrosine phosphatase-β (RPTPβ), neuronal contactin and contactin associated protein 1 (Caspr1). Once bound together, this family collectively contributes to glial-neuronal adhesiveness and to cell to cell communications [25–27]. The interaction between neuronal circuits and glial cells can be further influenced by metabolic signals, various environmental insults, and drugs of abuse. With regard to the latter, alcohol (ALC) is a drug of abuse that is known to alter hypothalamic functions that control reproduction. Chronic ALC exposure has been shown to cause depressed prepubertal LHRH secretion, and delayed pubertal development in both rodents [28–33] and primates [34,35]. This action of ALC is important since the onset of mammalian puberty is dependent upon an increase in the release of LHRH from the basal hypothalamus.

Recently, it has been suggested that some of the hypothalamic effects of ALC are due to ALC-induced interferences in glial-neuronal signaling networks involved in LHRH secretion. This review will mainly discuss two emerging glial-neuronal communication networks regarding their respective relationships to prepubertal LHRH secretion, and then address the actions and interactions of ALC on these glial-neuronal components during pubertal development.

## 2. EGF/TGFα Family of Growth Factors and Puberty

Growth factors of glial cell origin, acting via receptor tyrosine kinases, have been shown to be key components of the mechanism by which hypothalamic glial cells regulate LHRH neuronal function [36–38]. EGF and TGFα are peptides that signal through the erbB1 receptor. While both can stimulate LHRH release [9], TGFα has been shown to play a more pivotal role in the regulation of LHRH neuronal function during puberty in rodents [13,15] and primates [16]. TGFα mRNA and protein are highly expressed in glial cells and tanycytes in the MBH, their expressions increase significantly around the time of puberty [39], and pharmacological blockade of the erbB 1 receptor [40] targeted to the MBH delays puberty [39]. Additionally, sexual maturation induced by hypothalamic lesions is associated with activation of TGFα expression in glial cells [41,42], and the effect of this lesion on puberty is blocked by using a selective inhibitor (RG-50864) of TGFα/EGF receptor tyrosine kinase activity [41]. Also, advanced puberty has been observed in transgenic mice overexpressing the TGFα gene [43] and in rats carrying grafted cells genetically engineered to secrete TGFα [44]. TGFα binds to and activates the erbB1/erbB2 receptor complex on adjacent glial cells in MBH. Activation of these receptors results in the production and release of prostaglandin-E_2_ (PGE_2_) from these glial cells, which then induces prepubertal LHRH secretion upon binding to specific receptors on nearby LHRH neuron terminals in the median eminence (ME) region of the MBH [22,37]. This is supported by the fact that the stimulatory effect of TGFα on PGE_2_ release was blocked by administering an erbB1 receptor antagonist (RG-50864) [37]. Several studies have shown that TGFα stimulates LHRH release via an indirect mechanism that involves a paracrine effect of this growth factor on glial cells in the release of LHRH. In this regard, the erbB1 receptors for TGFα have been shown immunohistochemically only in glial cells [15,16]. Furthermore, Ma *et al.* [37] have shown *in vitro* that the secretion of PGE_2_ from hypothalamic glial cells is increased after exposure to TGFα and that the conditioned medium of hypothalamic glial cells treated with TGFα is able to stimulate LHRH release from GT1 cells, which are immortalized LHRH secreting neurons. Moreover, in hypothalamic glial cells, PGE_2_ formation induced by TGFα and the stimulatory effect of the TGFα treated conditioned medium on LHRH release are shown to be prevented by the inhibition of erbB receptor tyrosine kinase activity or prostaglandin synthesis [37,45]. Collectively, these data strongly support the notion that TGFα acts indirectly in the functional control of neuronal networks governing mammalian puberty via hypothalamic glial-neuronal communications.

## 3. Effects of ALC on the TGFα/erbB1 Receptor/PGE_2_ Pathway

**Figure 1:**
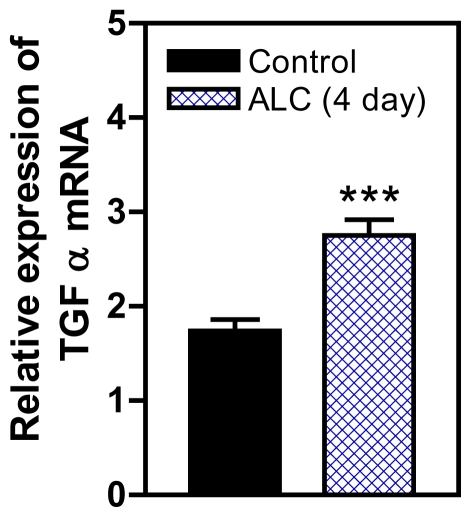
Effect of short-term ALC (ethanol) exposure on basal TGFα gene expression in the MBH of perpubertal female rats. Note that the ALC-treated animals showed an increase in the gene expression of TGFα compared with control animals. Values represent mean ± SEM. *N* = 12–14 animals per group; *** *p* < 0.001 *versus* control.

**Figure 2:**
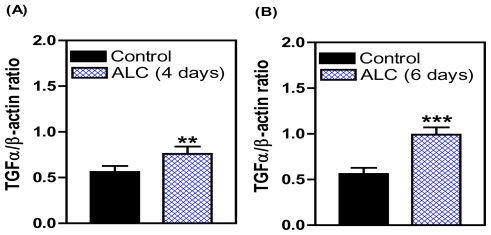
Effect of short-term ALC (ethanol) exposure on TGFα protein expression in the MBH of prepubertal female rats. Composite graphs that show the densitometric quantitation of the bands corresponding to TGFα protein. These data were normalized to the internal control β-actin protein, and the densitometric units represent the TGFα/β-actin ratio. Note that ALC caused an increase in TGFα protein expression on both 4 and 6 days compared with control animals. Values represent mean ± SEM. *N* = 7–8 per group. ** *p* < 0.01; *** *p* < 0.001 *versus* control.

**Figure 3:**
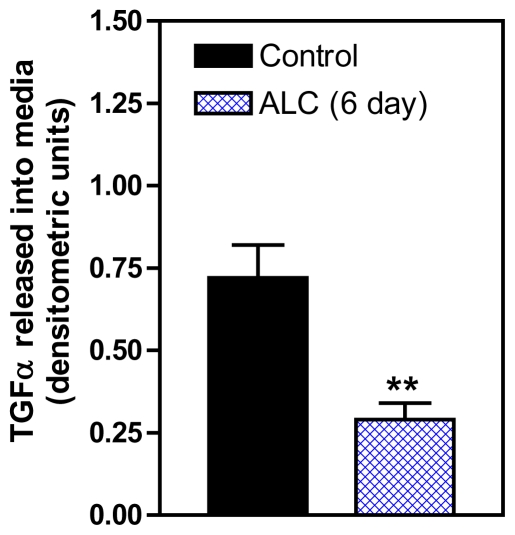
Effect of short-term *in vivo* ALC (ethanol) exposure for on TGFα protein released *in vitro* from the MBH of prepubertal female rats. Note that TGFα release was decreased in ALC-treated animals compared with control animals. These data were normalized to the internal control β-actin protein, and the densitometric units represent TGFα/β-actin ratio. Values represent mean ± SEM. *N* = 14 for control, *N* = 9 for ALC, ** *p* < 0.01 *versus* control.

It has been established that ALC acts within the hypothalamus to suppress the release of LHRH in both prepubertal and adult rats [46,47] and primates [35], and also causes delayed signs of pubertal maturation in both species [28,34]. Studies to discern the mechanism of this action of ALC to suppress LHRH release are important for understanding how this drug disrupts pubertal development. An important component of this ALC effect is PGE_2_, which plays a major role in the LHRH secretory process in prepubertal animals [48,49], and is known as a critical factor for glial-dependent regulation of LHRH release [21,37]. We showed previously [50] that acute ALC alters the EGF/TGFα-erbB1 receptor-COX (cyclooxygenase)-PGE_2_ pathway by inhibiting the induction of COX, the rate limiting enzyme necessary for prostaglandin synthesis and lowers prepubertal PGE_2_ secretion resulting in suppressed LHRH release [50,51]. Only recently have the mechanisms by which short-term ALC exposure affects the TGFα-erbB1 receptor -PGE_2_ pathway been assessed with regard to glial-neuronal communications within the prepubertal hypothalamus [52]. That study has revealed that short-term ALC exposure for 4 and 6 days caused an increase in TGFα gene and protein expressions in prepubertal female rats. The gene expression of TGFα was increased markedly at 4 days (Figure 1). After 6 days of ALC exposure, the level of TGFα gene expression was still modestly but significantly elevated; however, the levels had declined markedly (not shown) as compared to 4 days of exposure. This effect paralleled an increase in TGFα protein expression at both 4 days (Figure 2A) and 6 days (Figure 2B). To determine if the elevated hypothalamic levels of TGFα protein were due to an inhibition of release, we assessed basal TGFα secretion from rat MBHs incubated *in vitro* following 6 days of ALC exposure *in vivo*. We determined that animals exposed to ALC had suppressed release of TGFα (Figure 3). Overall, these findings suggest that ALC exposure does not affect transcription or translation of TGFα, but is capable of interfering with the hypothalamic release of glial TGFα, resulting in suppressed prepubertal PGE_2_ and LHRH secretion.

**Figure 4:**
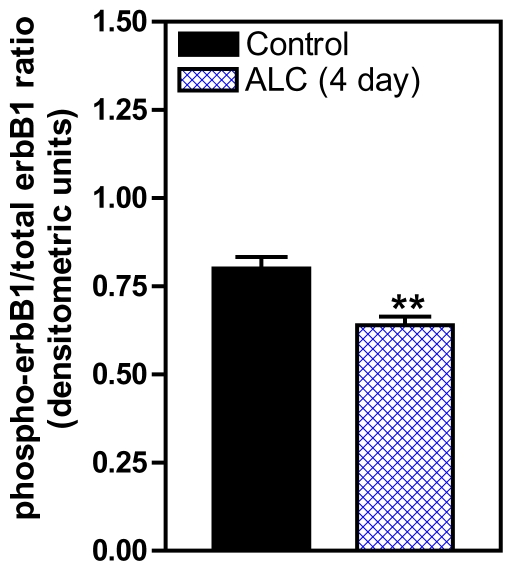
Effect of short-term ALC (ethanol) exposure on phosphorylated erbB1 protein expressions in the MBH of prepubertal female rats. Note that the ALC-treated animals showed a marked decrease in phosphorylated erbB1 protein expression compared with controls. These data were normalized to the total, nonphosphorylated erbB1 protein, and the densitometric units represent the phosphorylated erbB1/total, non-phosphorylated erbB1 ratio. Values represent mean ± SEM. *N* = 8 per group. ** *p* < 0.01 *versus* control.

This study also showed that the erbB1 receptor, the principal receptor for TGFα was affected by ALC. Short-term ALC exposure for 4 and 6 days caused a marked decrease in the synthesis of the phosphorylated form of the erbB1 receptor at 4 days (Figure 4), with 6 days being almost identical (not shown), but did not elicit changes in erbB1 gene expression or the synthesis of total, non-phosphorylated erbB1 protein. It is possible that down regulation of erbB1 gene synthesis had not yet occurred because of this short-term duration of ALC exposure, but it does appear ALC affected the phosphorylation of the erbB1 protein. Interestingly, in this study ALC did not affect the synthesis of total and phosphorylated erbB2, the co-receptor necessary for activation of erbB1 signaling [38] (not shown), which further demonstrates a specific effect of ALC on the erbB1 receptor. The mechanism of this action of ALC on erbB1, however, remains unclear. Since TGFα binding initiates the autophosphorylation of the erbB1 receptor [53,54], it is likely that the ALC-induced impairment of TGFα release (Figure 3) is a major contributing factor responsible for decreased erbB1 phosphorylation observed in the ALC-treated animals. However, we cannot rule out the possibility of a direct effect of ALC on the erbB1 autophosphorylation process that is independent of its effect to suppress TGFα secretion. In this regard, studies have demonstrated that chronic ALC exposure can disrupt phosphorylation of erbB1 by altering the receptor affinity and/or tyrosine kinase activity in rats [55,56].

**Figure 5:**
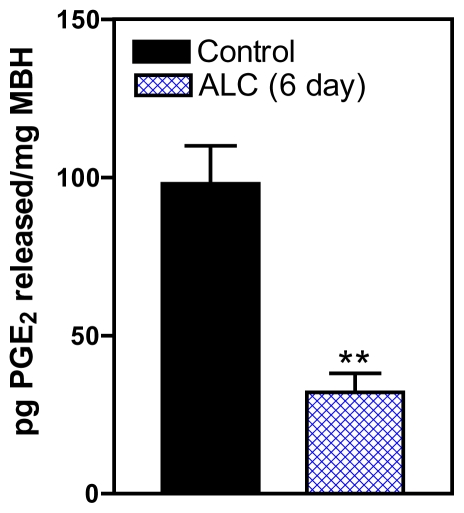
Effect of *in vivo* ALC (ethanol) exposure on prostaglandin-E_2_ (PGE_2_) release *in vitro* from the MBH of prepubertal rats as determined by enzyme-linked immunoassay. Note that the ALC-treated animals showed a marked decrease in basal PGE_2_ release compared with control animals. Values represent mean ± SEM. *N* = 8 for control, *N* = 9 for ALC. ** *p* < 0.01 *versus* control.

Since it was expected that suppressed erbB1 phosphorylation would result in a downstream decrease in PGE_2_ release, animals were chronically exposed to ALC for 6 days and then their hypothalami removed and incubated *in vitro* in order to assess the amount of PGE_2_ released into the incubation medium. This revealed that ALC exposure suppressed the release of PGE_2_ (Figure 5), an action that was associated with the suppressed phosphorylation of the erbB1 receptor shown in Figure 4. These findings demonstrated for the first time the upstream inhibitory effects of ALC on glial TGFα/erbB1 pathways that control the production and secretion of PGE_2_ within the MBH; hence, providing a mechanism supporting previous reports showing that ALC is capable of suppressing PGE_2_ and subsequently, LHRH secretion [50,51,57,58].

**Figure 6:**
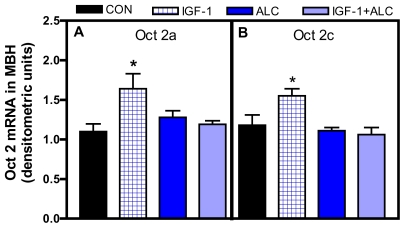
Effect of acute ALC (ethanol) exposure on IGF-1 induced Oct 2a and 2c in the MBH of prepubertal female rats. Panels A and B depict densitometric quantitation corresponding to the Oct 2a and 2c transcripts. IGF-1induced an increase in Oct2a and 2c mRNA over basal synthesis. Acute ALC exposure did not affect basal synthesis of Oct 2a and 2c mRNA but blocked the IGF-1 induced synthesis of both Oct 2a and 2c. *N* = 5–6/group. * *p* < 0.05.

Studies in recent years have identified upstream regulatory sites in the TGFα/erbB1/PGE_2_/LHRH-secretory pathway. Investigators have shown that the expression of the POU homeodomain gene, Oct2, increases in the MBH at puberty and that this increase was associated with the transactivation of TGFα gene expression [59]. Because IGF-1 plays an important role in control of prepubertal LHRH release, we investigated the possibility that IGF-1 may influence transcription of the Oct 2 gene and that this gene may be a target of ALC actions [60]. It was demonstrated that a single injection of IGF-1 to 25 day-old female rats caused increases in both Oct 2a and c gene transcripts in the MBH and furthermore, showed that an acute dose of ALC (3g/kg) did not alter basal expression of the gene transcripts, but blocked the IGF-1 induced expressions (Figure 6a and b). Similar effects were observed regarding Oct 2c in the POA, but not the Oct 2a transcript. Other investigators showed an inhibitory effect of chronic ALC exposure on Oct 2 proteins expressed in the entire rostro-caudal extent of the prepubertal hypothalamus [61].

**Figure 7:**
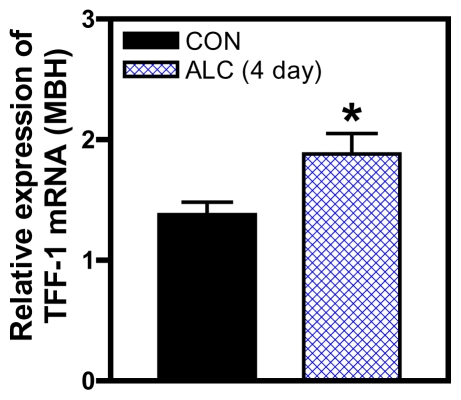
Effect of short-term ALC (ethanol) exposure on TTF1 mRNA expression from the MBH of prepubertal female rats. Note that TTF1 was increased in ALC-treated animals compared with control animals. Values represent mean ± SEM. *N* = 9 for control, *N* = 9 for ALC, * *p* < 0.05 *versus* control.

Interestingly, ALC exposure has also been shown to affect the gene encoding thyroid transcription factor 1 (TTF1). TTF-1 is a member of the Nkx homeodomain gene family that increases in the hypothalamus at puberty and has the ability to activate LHRH neurons [38,62]. It was shown that TTF1 expression peaked at postnatal day 26–27 in controls but that ALC administration beginning at 24 days of age caused suppressed hypothalamic TTF1 protein expression between 25 and 27 days, which was followed by a significant increase by 30 days [61]; thus, those authors suggested that the ALC delayed the peak increase in prepubertal TTF1 protein expression. Recently, we observed that ALC exposure beginning on 27 days of age was associated with an increase in TTF1 gene expression in the MBH (Figure 7) at 31 days. This increase at 31 days did not occur in the POA, however, by day 33, both hypothalamic regions showed elevated TTF1 gene expressions compared with controls (not shown). While additional research is needed in this area, this observation supports the earlier report by Kim *et al.* [61], that ALC postponed the prepubertal increase in TTF1 synthesis.

## 4. Adhesion/Signaling Genes Involved in Glial-Neuronal Communication during Puberty

There is growing evidence that glial cells can also regulate LHRH secretion by contributing to plastic arrangements associated with glial-neuronal adhesiveness [13]. Since adhesion/signaling molecules are abundant in the MBH, and have cell signaling actions [23,63], it has been suggested that they play a role in intracellular communication between glial cells and LHRH neuron terminals [64,65]. In this regard, several genes have been identified which synthesize adhesion/signaling proteins responsible for the structural integrity of bi-directional glial-neuronal communication. The synaptic cell adhesion molecule (SynCAM1) expressed by both glia and neuronal cells, promotes the glial-neuronal adhesiveness via homophylic, extracellular domain-mediated interactions required for synaptic assembly [13]. Similarly, another report using an *in vitro* adhesion assay shows that both hypothalamic glial cells and GT1-7 neuronal cells adhere to the extracellular domain of SynCAM1 and suggest the importance of this adhesion molecule in cell-cell communication between glial cells and LHRH neurons [64,65]. SynCAM1 is also associated with neuregulin activated erbB4 receptor, one of the glial cell erbB receptors involved in LHRH secretion at puberty [64,66] and is expressed in hypothalamic glial cells. Specifically, erbB4 receptor forms a heterodimer complex with erbB2 and activation by neuregulins causes LHRH release via an action involving glial PGE_2_ release and facilitation of erbB-1 mediated signaling events [66,67]. Disruption of erbB4/-2 signaling results in delayed puberty and an inhibition of SynCam1 expression in hypothalamic glial cells [66,67]. Futhermore, a study has shown that transgenic mice with a double negative form of SynCam1 lacking the intracellular domain causes these animals to have delayed puberty [68]. These observations suggest that one of the mechanisms underlying erbB4 receptor facilitation of LHRH neuronal function involves SynCAM1 dependent signaling during pubertal development. The effect of ALC exposure on SynCAM1 gene and protein expression is under investigation.

Another example among the adhesion/signaling genes within the MBH is a three member family consisting of neuronal contactin associated protein-1 (Caspr1), a transmembrane protein that binds to contactin on the same neuronal cell membrane. The contactin portion of this Caspr1/contactin complex is bound by a glial transmembrane protein, receptor protein tyrosine phosphatase beta (RPTPβ); thus, forming the three member assembly that can contribute to glial-neuronal adhesiveness [25,26]. Contactin participates in axonal growth, synaptogenesis and neuroendocrine function, and contactin expression in hypothalamic secretory neurons has been shown to be altered in response to changes in glial-neuronal associations [27]. Caspr1 is proposed as a signaling molecule of contactin, which mediates its cell adhesion by binding to RPTPβ and activating intracellular signaling pathways in neurons [63]; thus, following binding, this three member assembly contributes to bidirectional cell-cell communications between glial and neuronal cells associated with hypothalamic neuroendocrine functions [27]. It is important to note that LHRH neurons express both contactin and Caspr1 [23]. It has been suggested that upon binding together this system not only provides adhesiveness between glial connections with LHRH terminals, but also regulates intracellular processes [64]. Interestingly, the intimate association between glial “end feet” and LHRH neuron terminals with the ME area is modified by the actions of different reproductive factors [69–71], in that changes in the contact between glia and neurons fluctuates depending on steroid milieu and stage of pubertal devleopment [72]. Taken together, a concept is emerging that suggests that these glial-neuronal molecules facilitate hypothalamic neurosecretion via changes in the arrangement of glial-neuronal adhesion and signaling that could alter LHRH release and the pubertal process.

## 5. Effects of ALC on Glial-Neuronal Adhesion and Signaling

**Figure 8:**
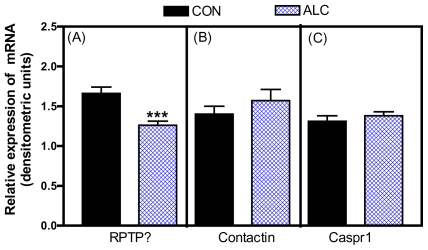
Effect of short-term ALC (ethanol) exposure on the gene expressions of basal RPTPβ (A), Contactin (B) and Caspr1(C) in the MBH of prepubertal female rats. Note that ALC caused a marked decrease in the gene expression of basal RPTPβ compared with control animals. Values represent mean ± SEM. *N* = 12–13 per group. *** *p* < 0.001 *versus* control.

**Figure 9:**
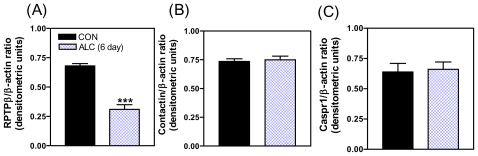
Effect of short-term ALC (ethanol) exposure on the protein expressions of basal RPTPβ (A), contactin (B) and Caspr1(C) in the MBH of prepubertal rats. Note that the protein expression of RPTPβ was markedly decreased in ALC exposed animal; however, the protein expressions of contactin and Caspr1 were unaltered. These data were normalized to the internal control β-actin protein, and the densitometric units represent the respective specific protein/β-actin ratio. Values represent mean ± SEM. *N* = 10 for control, *N* = 6 for ALC. *** *p* < 0.001 *versus* control.

In addition to ALC affecting neuronal inputs involved in prepubertal LHRH secretion, there has been some recent attention given to the actions of this drug on the contactin-Caspr1-RPTPß signaling system as it relates to glial-LHRH neuronal interactions [73]. In this regard, short-term ALC exposure caused a marked decrease in the basal expression of the RPTPβ gene (Figure 8A), but did not affect the expression of either contactin (Figure 8B) or Caspr1 (Figure 8C). Similarly, ALC caused suppressed levels of the RPTPβ protein (Figure 9A), with the expressions of both contactin (Figure 9B) and Caspr1 (Figure 9C) proteins being unaltered. The ALC-induced decrease in glial RPTPβ gene expression in this study indicates a reduced amount of peptide available for binding to the contactin-Caspr1 complex on LHRH neurons. As a result, decreased adhesiveness between the glia and LHRH neurons in the MBH following ALC exposure would be expected, an action that could affect neuronal functions associated with pubertal development. The binding of glial RPTPβ to the contactin/Caspr1 complex on neuron terminals initiates the cell adhesion and subsequently, activates neuronal intracellular signaling pathways [63]. The precise contribution of contactin-Caspr1 signaling to LHRH secretion remains to be determined, but previous studies have suggested that a function of the RPTPβ-contactin-Caspr1 family is to facilitate neurosecretion through changes in glial-neuronal signaling [23,27]. The cell adhesiveness component also promotes a more secure proximity for glial-derived secretions to bind to their receptors on the LHRH nerve terminals. Whatever the mechanism, the fact that ALC alters the synthesis of prepubertal glial RPTPβ in the hypothalamus, which is required for binding to the neuronal contactin/Caspr1 complex, suggests diminished glial-neuronal adhesiveness and thus, altered facilitation of LHRH release by the products secreted from the neighboring glial cells.

## 6. Actions and Interactions of IGF-1 and ALC on Glial-Neuronal Adhesion and Signaling

**Figure 10:**
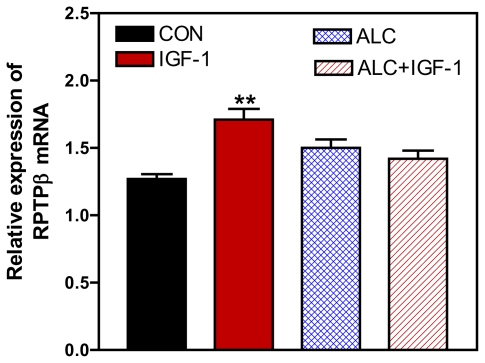
Effect of acute ALC exposure on basal and IGF-1 stimulated gene expressions of RPTPß in the MBH of prepubertal female rats. Note that IGF-1 increased the gene expression of RPTPβ compared with the basal level of control. Importantly, the basal expression of the RPTPβ gene was not altered by ALC alone, but the IGF-1 induced expression of RPTPβ was blocked by the presence of ALC. Values represent mean ± SEM. *N* = 8–10 per group. ** *p* < 0.01 *versus* control and ALC + IGF-1.

IGF-1 plays a critical role in the pubertal process. Initially, it was shown that the peptide is capable of inducing release of LHRH from the prepubertal hypothalamus *in vitro* [10], then later demonstrated thast IGF-1 acts within the hypothalamus to accelerate the timing of female puberty [11]. Subsequently, it was show that premature elevation of serum IGF-1 advanced first ovulation in rhesus monkeys [74]. While IGF-1 is produced by neurons and glia in the MBH, the majority of IGF-1 present in this region during puberty is derived from peripheral sources, such as liver [11,75,76]. IGF-1 binds to type 1 IGF receptors (IGF-1R) in different tissues including the brain, where the greatest concentration of IGF-1R has been observed in the ME of both rats [77] and primates [78]. Recently, DiVall *et al.* [79] showed that transgenic mice lacking the IGF-1R on their LHRH neurons have delayed puberty, further supporting the importance of this peptide for pubertal development. IGF-1 is also capable of acting at both glial and neuronal levels involved in neuroendocrine events within the hypothalamus in association with prepubertal LHRH secretion [47]. These findings suggest that IGF-1 may play a role in regulating the expression of adhesion and signaling molecules. Interestingly, we showed that IGF-1 induced an increase in the expression of the RPTPß gene in prepubertal female rat hypothalamus (Figure 10); however, it did not affect the gene expression of either contactin or Caspr1 [73]. Glial RPTPß is expressed abundantly within the MBH [80]. The knowledge that increased circulating levels of IGF-1 at puberty can cross the blood brain barrier and enter the MBH region [11], and that the circulating peptide is taken up by hypothalamic glia in the MBH [81], suggests that the IGF-1-induced expression of RPTPß in the MBH may facilitate neurosecretion via changes in glial-neuronal adhesive signaling.

The fact that IGF-1 is important for LHRH release at puberty [10,11] and the observation that ALC can alter prepubertal IGF-1 signaling [29] suggests that ALC could have a detrimental impact on pubertal development by interfering with glial neuronal signaling networks. In support of this, it has been shown recently [73] that ALC blocked the ability of IGF-1to induce prepubertal RPTPβ gene expression (Figure 10). The mechanism by which ALC influenced the IGF-1 induced expression of RPTPβ remains to be determined; however, this effect could be due to an action of ALC at the level of the IGF-1R and/or to an altered post-receptor event. Interestingly, ALC administration does not alter IGF-1R gene or protein expression [29], although we cannot rule out that it may affect mechanisms regulating receptor function. This suggests that ALC may act on a pathway component downstream from the IGF-1R. In this regard, chronic ALC administration was shown to suppress the basal protein expression of phosphorylated Akt, a transduction signal activated by IGF-1 [82]. Acutely, ALC was shown to block the IGF-1 induced expression of other genes involved in the pubertal process [60,83], and that this occurs by blocking the peptides ability to induce phosphorylation of Akt [83]. Chronic exposure to ALC has been shown to decrease circulating levels of IGF-1 in prepubertal rats [29] and rhesus monkeys [34]; actions associated with altered pubertal development. Based on these collective results, it is suggested that the decreased prepubertal levels of circulating IGF-1 available to the MBH, as well as altered post-receptor transduction signals, may contribute to the ability of ALC to cause the suppression in RPTPβ gene expression.

## 7. Conclusions

**Figure 11:**
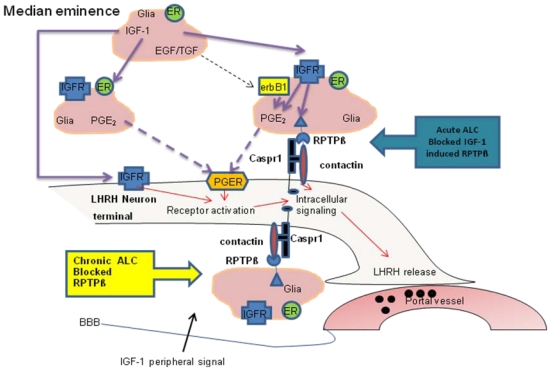
Schematic drawing showing glial-LHRH neuronal associations and sites of ALC effects on RPTPβ in the median eminence of juvenile female rats. For clarity, details of other downstream pathways in this region are not shown. ALC, alcohol; BBB, blood brain barrier; Caspr1, contactin associated protein-1; EGF, epidermal growth factor; erbB1, EGF/TGF receptor; ER, estrogen receptor; IGF-1, insulin-like growth factor-1; IGFR, Insulin-like growth factor-1 receptor; LHRH, luteinizing hormone releasing hormone; PGE_2_, prostaglandin-E_2_; PGER, prostaglandin-E_2_ receptor; RPTPβ, receptor protein tyrosine phosphatase β; TGF, transforming growth factor [73].

It is becoming increasingly clear that glial-neuronal interactions play an important role in prepubertal LHRH secretion and the subsequent acquisition of female pubertal development. In this review, we have mainly discussed the actions of glial-neuronal communication networks at puberty and how these actions are influenced by ALC, a drug of abuse known to alter pubertal development. A summary of these cellular communications and the sites of ALC actions are shown in Figure 11. One of these communication systems is the TGFα-erbB1 receptor signaling system. We have made reference to research indicating that glial TGFα activates the erbB1/erbB2 receptor complex on adjacent glia in the MBH. This activation causes a cascade of events leading to the increased synthesis and release of PGE_2_, which then binds to its receptor on nearby LHRH neuron terminals causing stimulated release of the peptide. Additionally, evidence was presented showing that ALC is capable of interfering with hypothalamic glial to glial signaling involved with prepubetal PGE_2_ sythesis/release. The other communication network discussed in detail is the contactin-Caspr1 complex that binds to glial RPTPβ. After binding together, the newly formed three member family provides adhesiveness and promotes signaling between glia and LHRH nerve terminals in the MBH. Interestingly, we provided evidence that ALC exposure can interfere with this glial-neuronal communication family at puberty by suppressing the synthesis of glial RPTPβ. Additionally, we discussed how IGF-1 can influence both PGE_2_ and RPTPβ signaling. Overall, this review further indicates that glial-neuronal communications are important for LHRH secretion at puberty, and that ALC is capable of altering these cell-cell interactions. There are obviously other glial-neuronal systems not discussed here that deserve further investigation into their respective roles in neuroendocrine secretion at puberty, as well as determining whether they are affected by endocrine disruptive substances that may alter their functions.

## References

[1] Brann DW, Mahesh VB (1994). Excitatory amino acids: Function and significance in reproduction and neuroendocrine regulation. Front. Neuroendocrinol.

[2] Ojeda SR, Urbanski HF, Knobil E, Neill JD (1994). Puberty in the rat. The Physiology of Reproduction.

[3] Crowley WR, Parker SL, Sahu A, Kalra SP, Plant TM, Lee PA (1995). Interacting transmembrane signals regulating GnRH and LH secretion. The Neurobiology of Puberty.

[4] Ojeda SR (1994). The neurobiology of mammalian puberty: Has the contribution of glial cells been underestimated?. J. NIH Res.

[5] Terasawa E (1999). Hypothalamic control of the onset of puberty. Curr. Opin. Endocrinol. Diabetes.

[6] Terasawa E, Fernandez DL (2001). Neurobiological-mechanisms of the onset of puberty in primates. Endocr. Rev.

[7] Claypool LE, Kasuya E, Saitoh Y, Marzban F, Terasawa E (2000). *N*-methyl-d,l-aspartate induces the release of LHRH in the prepubertal and pubertal female rhesus monkey as measured by in vivo push-pull perfusion in the stalk-median eminence. Endocrinology.

[8] Urbanski HF, Ojeda SR (1990). A role for *N*-methyl-d-aspartate (NMDA) receptors in the control of LH secretion and initiation of female puberty. Endocrinology.

[9] Ojeda SR, Urbanski HF, Costa ME, Hill DF, Moholt-Siebert M (1990). Involvement of transforming growth factor alpha in the release of luteinizing hormone-releasing hormone from the developing female hypothalamus. Proc. Natl. Acad. Sci. USA.

[10] Hiney JK, Ojeda SR, Dees WL (1991). Insulin-like growth factor (IGF-1) stimulates LHRH release from the prepubertal female median eminence in vitro. Neuroendocrinology.

[11] Hiney JK, Srivastava VK, Nyberg CL, Ojeda SR, Dees WL (1996). Insulin-like growth factor-1 (IGF-1) of peripheral origin acts centrally to accelerate the initiation of female puberty. Endocrinology.

[12] Navarro CL, Castellano JM, Fernandez-Fernandez R, Barriero ML, Roa J, Sanhez-Criado JE, Aguilar E, Dieguez C, Pinilla L, Tena-Sempere M (2004). Developmental and hormonally regulated mRNA expression of KiSS-1 and its putative receptor, GPR54, in rat hypothalamus and potent LH-releasing activity of KiSS-1 peptide. Endocrinology.

[13] Ojeda SR, Lomniczi A, Sandau US (2008). Glial-gonadotropin hormone (GnRH) neuron interactions in the median eminence and the control of GnRH secretion. J. Neuroendocrinol.

[14] Ojeda SR, Lomniczi A, Sandau U (2010). Contribution of glial-neuronal interactions to the neuroendocrine control of female puberty. Eur. J. Neurosci.

[15] Ma YJ, Berg-von der Emde K, Moholt-Siebert M, Hill DF, Ojeda SR (1994). Region specific regulation of transforming growth factor-α (TGFα) gene expression in astrocytes of the neuroendocrine brain. J. Neurosci.

[16] Ma YJ, Costa ME, Ojeda SR (1994). Developmental expression of the genes encoding transforming growth factor alpha and its receptor in the hypothalamus of female rhesus macaques. Neuroendocrinology.

[17] Tsai PS, Werner S (1995). Basic fibroblast growth factor is a neurotropic factor in GT1 gonadotropin-releasing hormone neuronal cell lines. Endocrinology.

[18] Voigt P, Ma YJ, Gonzales D, Fahrenbach WH, Wetsel WC, Berg-Von der Emde K, Hill DF, Taylor KG, Costa ME, Seidah NG, Ojeda SR (1996). Neural and glial mediated effects of growth factors acting via tyrosine kinase receptor on LHRH neurons. Endocrinology.

[19] Prevot V (2002). Glial-neuronal-endothelial interactions are involved in the control of GnRH secretion. J. Neuroendocrinol.

[20] Olson BR (1995). Effects of insulin-like growth factors I and II and insulin on the immortalized hypothalamic GT1-7 cell line. Neuroendocrinology.

[21] Prevot V, Cornea A, Mungenast A, Smiley G, Ojeda SR (2003). Activation of erb-1 signaling in tanycytes of the median eminence stimulates transforming growth factor β1 release via prostaglandin E2 production and induces cell plasticity. J. Neurosci.

[22] Rage F, Lee BJ, Ma YJ, Ojeda SR (1997). Estradiol enhances prostaglandin E2 (PGE2) receptor gene expression in luteinizing hormone-releasing hormone (LHRH) neurons and facilitates the LHRH response to PGE2 by activating a glia-to-neuron signaling pathway. J. Neurosci.

[23] Mungenast AE, Ojeda SR (2005). Expression of three gene families encoding cell-cell communication molecules in the prepubertal nonhuman primate hypothalamus. J Neuroendocrinol.

[24] Ojeda SR, Dubay C, Lomniczi A, Kaidar G, Matagne V, Sandau US, Dissen GA (2010). Gene networks and the neuroendocrine regulation of puberty. Mol. Cell. Endocrinol..

[25] Peles E, Nativ M, Campbell PL, Sakuria T, Martinez R, Lev S, Clary DO, Schilling J, Barnea G, Plowman GD, Gurmet M, Schlessinger J (1995). The carbonic anhydrase domain of receptor tyrosine phosphatase ß is a functional ligand for the recognition molecule contactin. Cell.

[26] Peles E, Schlessinger J, Grumet M (1998). Multi-ligand interactions with receptor-like protein tyrosine phosphatase β: implications for intercellular signaling. Trends Biochem Sci.

[27] Pierre K, Rougon G, Allard M, Bonhomme R, Gennarini G, Poulain DA, Theodosis DT (1998). Regulated expression of the cell adhesion glucoprotein F3 in adult hypothalamic magnocellular neurons. J. Neurosci.

[28] Dees WL, Skelley CW (1990). The effects of ethanol during the onset of female puberty. Neuroendocrinology.

[29] Srivastava VK, Hiney JK, Nyberg CL, Dees WL (1995). Effect of ethanol on the synthesis of insulin-like growth factor-1(IGF-1) and the IGF-1 receptor in late prepubertal female rats: a correlation with serum IGF-1. Alc. Clin. Exp. Res.

[30] Srivastava VK, Hiney JK, Dearth RK, Dees WL (2002). Chronic effects of prepubertal ethanol administration on steroidogenic acute regulatory protein in the rat ovary. Alc. Clin. Exp. Res.

[31] Emanuele N, Ren J, LaPaglia N, Steiner J (2002). EtOH disrupts female mammalian puberty: age and opiate dependence. Endocrine.

[32] Anderson RA, Willis BR, Oswald C, Gupta A, Zaneveld L (1981). Delayed male sexual maturation induced by chronic ethanol ingestion. Fed. Proc.

[33] Ramaley JA (1982). The regulation of gonadotropin secretion in immature ethanol-treated male rats. J. Androl.

[34] Dees WL, Dissen GA, Hiney JK, Lara F, Ojeda SR (2000). Alcohol ingestion inhibits the increased secretion of puberty-related hormones in the developing female rhesus monkey. Endocrinology.

[35] Dissen GA, Dearth RK, Scott HM, Ojeda SR, Dees WL (2004). Alcohol alters prepubertal luteinizing hormone secretion in immature female rhesus monkeys by a hypothalamic action. Endocrinology.

[36] Cardona-Gomez GP, Doncarlos L, Garcia-Segura LM (2000). Insulin-like growth factor I receptors and estrogen receptors colocalize in female rat brain. Neuroscience.

[37] Ma YJ, Berg-von der Emde K, Rage F, Wetsel WC, Ojeda SR (1997). Hypothalamic astrocytes respond to transforming growth factor alpha with the secretion of neuroactive substances that stimulate the release of luteinizing hormone-releasing hormone. Endocrinology.

[38] Ojeda SR, Ma YJ, Lee BJ, Prevot V (2000). Glia to neuron signaling and the neuroendocrine control of female puberty. Recent Prog. Horm. Res.

[39] Ma YJ, Junier MP, Costa ME, Ojeda SR (1992). Transforming growth factor α (TGFα) gene expression in the hypothalamus is developmentally regulated and linked to sexual maturation. Neuron.

[40] Yaish P, Gazit A, Gilon C, Levitzki A (1988). Blocking of EGF-dependent cell proliferation by EGF receptor kinase inhibitors. Science.

[41] Junier MP, Ma YJ, Costa ME, Hoffman G, Hill DF, Ojeda SR (1991). Transforming growth factor α contributes to the mechanism by which hypothalamic injury induces precocious puberty. Proc. Natl. Acad. Sci. USA.

[42] Junier MP, Hill DF, Costa ME, Felder S, Ojeda SR (1993). Hypothalamic lesions that induce female precocious puberty activate glial expression of the epidermal growth factor receptor gene: differential regulation of alternatively spliced transcripts. J. Neurosci.

[43] Ma YJ, Dissen GA, Merlino G, Coquelin A, Ojeda SR (1994). Overexpression of a human transforming growth factor-α (TGFα) transgene reveals a dual antagonistic role of TGFα in female sexual development. Endocrinology.

[44] Rage F, Hill DF, Sena-Esteves M, Breakfield XO, Coffey RJ, Costa ME, McCann SM, Ojeda SR (1997). Targeting transforming growth factor α expression to discrete loci of the neuroendocrine brain induces female sexual precocity. Proc. Natl. Acad. Sci. USA.

[45] Ojeda SR, Ma YJ (1999). Glial-neuronal interactions in the neuroendocrine control of mammalian puberty: facilitatory effects of gonadal steroids. J. Neurobiol.

[46] Dees WL, Hiney JK, Nyberg CL, Sarkar DK, Barnes CD (1995). Effects of ethanol on the reproductive neuroendocrine axis of prepubertal and adult female rats. The Reproductive Neuroendocrinology of Aging and Drug Abuse.

[47] Dees WL, Srivastava VK, Hiney JK (2009). Actions and interactions of alcohol and insulin-like growth factor-1 on female pubertal development. Alc. Clin. Exp. Res.

[48] Ojeda SR, Urbanski HF, Katz KH, Costa ME, Conn PM (1986). Activation of two different but complementary biochemical pathways stimulates release of hypothalamic luteinizing hormone releasing hormone. Proc. Natl. Acad. Sci. USA.

[49] Ojeda SR, Urbanski HF, Katz KH, Costa ME (1988). Prostaglandin E2 releases luteinizing hormone releasing hormone for the female juvenile hypothalamus through Ca^2+^ dependent, calmodulin independent mechanism. Brain Res.

[50] Hiney JK, Dearth RK, Srivastava V, Rettori V, Dees WL (2003). Actions of ethanol on epidermal growth factor receptor activated luteinizing hormone secretion. J. Stud. Alcohol.

[51] Hiney JK, Dees WL (1991). Ethanol inhibits LHRH release from the median eminence of prepubertal female rats in vitro: investigation of its action on norepinephrine and prostaglandin E2. Endocrinology.

[52] Srivastava VK, Hiney JK, Dees WL (2011). Prepubertal ethanol exposure alters hypothalamic transforming growth factor α and erbB1 receptor signaling in the female rat. Alcohol.

[53] Carpenter G, Wahl MI, Sporn MB, Roberts AB (1990). The epidermal growth factor family. Peptide Growth Factors and their Receptors.

[54] Ebner R, Derynck R (1991). Epidermal growth factor and transforming growth factor α: Differential intracellular routing and processing of ligand-receptor complexes. Cell Regul.

[55] Tuma DJ, Todero SL, Barak-Bernihagen M, Casey CA, Sorrell MF (1998). Chronic ethanol ingestion impairs TGf α stimulated receptor autophosphorylation. Alcohol.

[56] Wang S, Feng J, Ying C, Wang W (1997). Time-dependent alteration of epidermal growth factor receptor in rat stomach by ethanol feeding. Toxicol. Lett.

[57] Nyberg CL, Hiney JK, Minks JE, Dees WL (1993). Ethanol alters *N*-methyl-dl-Aspartic acid-induced LH secretion of luteinizing hormone releasing hormone and the onset of puberty in the female rats. Neuroendocrinology.

[58] Lomniczi A, Mastronardi CA, Faletti AG, Seilicovich A, De Laurentiis A, McCann SM, Rettori V (2000). Inhibitory pathways and the inhibition of luteinizing hormone-releasing hormone release by alcohol. Proc. Natl. Acad. Sci. USA.

[59] Ojeda SR, Hill J, Hill DF, Costa ME, Tapia V, Cornea A, Ma YJ (1999). The Oct-2 POU domain gene in the neuroendocrine brain: A transcriptional regulator of mammalian puberty. Endocrinology.

[60] Dees WL, Srivastava VK, Hiney JK (2005). Alcohol alters IGF-1 activated Oct-2 POU gene expression in the immature female hypothalamus. J. Stud. Alc.

[61] Kim HJ, Sohn HJ, Ha M, Han JY, Kang SS, Choi WS, Cho JG (2005). Prepubertal chronic ethanol administration alters TTF-1 and Oct-2 expression in the hypothalamus of female rats. Mol. Brain Res.

[62] Lee BJ, Cho GJ, Norgren RB, Junier MP, Hill DF, Tapia V, Costa ME, Ojeda SR (2001). TTF-1, a homeodomain gene required for diencephalic morphogenesis, is postnatally expressed in the neuroendocrine brain in a developmentally regulated and cell-specific fashion. Mol. Cell Biol..

[63] Peles E, Native M, Lustig M, Grumet M, Schilling J, Martinez R, Plowman GD, Schlissinger J (1997). Identification of a novel contactin-associated transmembrane receptor with multiple domains implicated in protein-protein interactions. EMBO J.

[64] Lomniczi A, Ojeda SR, Purpura V, Haydon P (2009). A role for glial cells of the neuroendocrine brain in the central control of female sexual development. Astrocytes in Pathophysiology of the Nervous System.

[65] Sandau US, Mungenast AE, McCarthy J, Biederer T, Corfas G, Ojeda SR (2011). The synaptic cell adhesion molecule, SynCAM1, mediates astrocyte-to astrocyte and astrocyte-to-GnRH neuron adhesiveness in the mouse hypothalamus. Endocrinology.

[66] Ma YJ, Hill DF, Creswick KE, Costa ME, Cornea A, Lioubin MN, Plowman GD, Ojeda SR (1999). Neuregulins signaling via a glial erb-2-erb-4 receptor complex contribute to the neuroendocrine control of mammalian sexual development. J. Neurosci.

[67] Prevot V, Rio C, Cho GJ, Lomniczi A, Heger S, Neville CM, Rosenthal NA, Ojeda SR, Corfas G (2003). Normal female sexual development requires neuregulin-erbB receptor signaling in hypothalamic astrocytes. J. Neurosci.

[68] Sandau US, Mungenast AE, Alderman Z, Pablo Sardi S, Fogel AI, Taylor B, Parent A, Biederer T, Corfas G, Ojeda SR (2011). SynCAM1, a synaptic adhesion molecule, is expressed in astrocytes and contributes to erbB4 receptor-mediated control of female sexual development. Endocrinology.

[69] King JC, Letourneau RL (1994). Luteinizing hormone releasing hormone terminals in the median eminence of rats undergo dynamic changes after gonadectomy, as revealed by electron microscopic image analysis. Endocrinology.

[70] Prevot V, Bouret S, Croix D, Alonso G, Jennes L, Mitchell V, Routtenberg A, Beayvillain JC (2000). Growth associated protein-43 messenger ribonucleic acid expression in gonadotropin releasing hormone neurons during the rat estrous cycle. Endocrinology.

[71] Theodosis DT, Poulain DA (1992). Neuronal-glial and synaptic plasticity of the adult oxytocinergic system. Ann. N. Y. Acad. Sci.

[72] Witkin JW, O’Sullivan H, Ferin M (1995). Glial ensheathment of GnRH neurons in prepubertal female rhesus macaques. J. Neuroendocrinol.

[73] Srivastava VK, Hiney JK, Dees WL (2011). Hypothalamic actions and interactions of alcohol and IGF-1 on the expression of glial receptor protein tyrosine phosphatase-ß during female pubertal development. Alc Clin Exp Res.

[74] Wilson ME (1998). Premature elevation in serum insulin-like growth factor-1 advances first ovulation in rhesus monkeys. J. Endocrinol.

[75] Handelsman DJ, Spaliviero JA, Scott CD, Baxter RC (1987). Hormonal regulation of the peripubertal stage of insulin-like growth factor-I in the rat. Endocrinology.

[76] Copeland KC, Kuehl TJ, Castracane VD (1982). Pubertal endocrinology of the baboon: elevated somatomedin-C/insulin-like growth factor I at puberty. J. Clin. Endocrinol. Metab.

[77] Bondy C, Werner H, Roberts CT, Leroith D (1992). Cellular pattern of type-I insulin-like growth factor receptor gene expression during maturation of the rat brain: comparison with insulin-like growth factors I and II. Neuroscience.

[78] Vendola K, Zhou J, Wang J, Bondy CA (1999). Androgens promote insulin-like growth factor-1 and insulin-like growth factor-1 receptor gene expression in the primate ovary. Hum. Reprod.

[79] DiVall SA, Williams TR, Carver SE, Koch L, Bruning JC, Kahn CR, Wondisford F, Radovick S, Wolfe A (2010). Divergent roles of growth factors in the GnRH regulation of puberty in mice. J. Clin. Invest.

[80] Parent AS, Mungenast AE, Lomniczi A, Sandau US, Peles E, Bosch MA, Ronnekliev OK, Ojeda SR (2007). A contactin receptor-like protein tyrosine phosphatase beta complex mediates adhesive communication between astroglial cell and gonadotropin releasing hormone neurons. J. Neuroendocrinol.

[81] Duenas M, Luquin S, Chowen JA, Torres-Aleman I, Naftolin F, Garcia-Segura LM (1994). Gonadal hormone regulation of insulin-like growth factor-I like immunoreactivity in hypothalamic astroglia of developing and adult rats. Neuroendocrinology.

[82] Srivastava VK, Hiney JK, Dees WL (2009). Short term alcohol administration alters KiSS-1 gene expression in the reproductive hypothalamus of prepubertal female rats. Alcohol. Clin. Exp. Res.

[83] Hiney JK, Srivastava VK, Dees WL (2010). Insulin-like growth factor-1 stimulates hypothalamic KiSS-1 gene expression by activating Akt: Effect of alcohol. Neuroscience.

